# Cardiomyopathies and Arrhythmias Induced by Cancer Therapies

**DOI:** 10.3390/biomedicines8110496

**Published:** 2020-11-12

**Authors:** Dragoș-Mihai Romitan, Dan Rădulescu, Ioana Berindan-Neagoe, Laurențiu Stoicescu, Alin Grosu, Liliana Rădulescu, Diana Gulei, Tudor-Eliade Ciuleanu

**Affiliations:** 1 Department of Cardiology, Municipal Clinical Hospital of Cluj-Napoca, 400139 Cluj-Napoca, Romania; office@spitalclujana.ro (D.R.); stoicescul@yahoo.com (L.S.); alin_grosu@yahoo.com (A.G.); lili_m_radulescu@yahoo.com (L.R.); 2 Research Center for Functional Genomic, Biomedicine and Translational Medicine, “Iuliu Hațieganu” University of Medicine and Pharmacy, 400139 Cluj-Napoca, Romania; genomica@umfcluj.ro; 3 Research Center for Advanced Medicine-Medfuture, “Iuliu Hațieganu” University of Medicine and Pharmacy Cluj-Napoca, 400139 Cluj-Napoca, Romania; diana.c.gulei@gmail.com; 4 Department of Chemotherapy, Ion Chiricuta Clinical Cancer Center, 400139 Cluj Napoca, Romania; tudor_ciuleanu@hotmail.com

**Keywords:** cardiotoxicity, chemotherapy, immunotherapy, cardiomyocytes, cardiomyopathy, arrhythmias

## Abstract

Cardiology and oncology are two fields dedicated to the study of various types of oncological and cardiac diseases, but when they collide, a new specialty is born, i.e., cardio-oncology. Continuous research on cancer therapy has brought into the clinic novel therapeutics that have significantly improved patient survival. However, these therapies have also been associated with adverse effects that can impede the proper management of oncological patients through the necessity of drug discontinuation due to life-threatening or long-term morbidity risks. Cardiovascular toxicity from oncological therapies is the main issue that needs to be solved. Proper knowledge, interpretation, and management of new drugs are key elements for developing the best therapeutic strategies for oncological patients. Upon continuous investigations, the profile of cardiotoxicity events has been enlarged with the inclusion of myocarditis upon administration of immune checkpoint inhibitors and cardiac dysfunction in the context of cytokine release syndrome with chimeric antigen receptor T cell therapy. Affinity enhanced and chimeric antigen receptor T cells have both been associated with hypotension, arrhythmia, and left ventricular dysfunction, typically in the setting of cytokine release syndrome. Therefore, the cardiologist must adhere to the progressing field of cancer therapy and become familiar with the adverse effects of novel drugs, and not only the ones of standard care, such as anthracycline, trastuzumab, and radiation therapy. The present review provides essential information summarized from the latest studies from cardiology, oncology, and hematology to bring together the three specialties and offers proper management options for oncological patients.

## 1. Introduction

Cardiovascular diseases and cancer are the best-known causes of mortality worldwide, cumulating 46.1% of the total number of deaths. Evidence-based studies have shown that the incidence of cancer-related diseases has grown and the cancer-related mortality has decreased, demonstrated by the expansion of the number of survivors of cancer since the 1990s [1,2]. The survival of patients is not only dependent on the tumor inhibitory efficiency of the treatments but also on knowledge about the adverse effects of neoplastic treatments that have a major role in the therapeutic management of patients, especially in the establishment of proper dosages. Pre-existing cardiovascular diseases can drastically influence therapy and can lead to the ending of cancer therapy. Cancer therapeutic-related dysfunctions were first discovered over 50 years ago, when scientists studied the risk of cardiomyopathy development in response to anthracyclines [3]. Since then, cancer therapies, from anthracyclines to immunotherapies and radiotherapy, have been associated with myocyte damage, left ventricular systolic and diastolic dysfunctions, congestive heart failure, cardiac arrhythmias, thrombosis, pericardial disease, hypertension, vasospasm, and myocardial ischemia [4,5]. Therefore, the evaluation and management of potential cardiovascular-related adverse effects in cancer patients receiving therapy is equally important as the therapeutic itself.

In this review, we describe three types of cardiomyopathy, i.e., primary (toxic) cardiomyopathy, referred to as cancer therapy-related type I and characterized by direct cardiomyocyte damage; secondary cardiomyopathy, referred to as cancer therapy-related type II in respect to alterations in perfusion, innervation, or modifications at the hormonal level; and type III cardiomyopathy, simply known as myocarditis that describes inflammatory cell infiltration in the myocardial cells.

## 2. Primary Cardiomyopathies

Cancer therapy-related type I cardiomyopathies can be observed in many forms of neoplastic treatments. These adverse effects intervene due to the direct toxic events caused by cancer treatment regimens on the myocardium and are the most frequent form of toxic cardiomyopathies [6]. Standard chemotherapeutics are compounds designed to target malignant cells and inhibit their viability by interfering with their mitotic activity. Among these, anthracyclines are one of the most recognized examples that inhibit DNA or RNA synthesis by direct interruption of DNA and RNA base pairs strands [7]. The mechanisms of anthracycline-related cardiotoxicity have a special characteristic. For example, anthracycline drugs inhibit topoisomerase IIα and target mitochondria in cardiomyocytes. Typical pathological changes in the heart include vacuolar degeneration of the sarcoplasmic reticulum, swelling and disruption of the mitochondria, and myofilament degeneration [8]. There is also evidence of myocyte loss. Pathologic evidences include early studies showing chromatin condensation, as can be seen in apoptotic cells in the myocardium of anthracycline-treated patients [9]. Mitochondrial injury is the main result of anthracycline-related cardiotoxicity [10]. There are also preclinical hypotheses that account ion dysregulation and changes in the cardiac profile of gene expression to be responsible for installation of cardiotoxicity upon chemotherapy [11]. The identification of specific molecular alterations that mediate the cardiotoxic events could sustain the future development of targeted drugs able to prevent the installation of such events. For example, iron chelators are among the first used cardioprotective agents for limitation of anthracyclines-induced oxidative stress through restoration of iron balance at the cellular level or through elimination of redox-active iron [12].

Cardiotoxicity, the disease caused mainly by dose-adverse effects to different types of therapies, including neoplastic therapies, is divided in two pathological forms, i.e., acute and chronic. The acute form is associated with more secondary effects than the chronic form, with ECG changes such as arrhythmias (up to 20–30% of patients), sinus tachycardia, supraventricular tachycardia, heart block, and ventricular arrhythmias (up to 3% of patients). Another major symptom that can appear is dyspnea with acute heart failure. Nevertheless, some patients can develop chest pain before shortness of breath or pericarditis [10,13,14].

Another example is trastuzumab, which is a humanized antibody directed against HER2 (also known as ERBB2) and which is overexpressed in 15–20% of breast cancers. On the one hand, HER2 signaling increases the development of tumors such as cell proliferation, tumor growth, and metastatic spread. On the other hand, inhibition of the HER2 pathway concludes in clinical success [15,16,17]. As in the case of anthracycline-related cardiotoxicity, cardiac function and heart failure are adverse effects which have been recognized in approximately 30% of patients in early clinical trials [18]. However, not long after the attempt of HER2 manipulation for the treatment of breast cancer, it was also discovered that HER2 signaling was also vital for cardiac physiology. This was highlighted by the installation of synergistic cardiac dysfunction effects in patients receiving trastuzumab or trastuzumab combined with doxorubicin [19]. Anthracyclines can induce myocardial oxidative stress, which can lead to myocardial damage. Trastuzumab increased myofibrillar disarray in anthracycline-treated rat cardiomyocytes, suggesting that trastuzumab enhanced the susceptibility of cardiomyocytes to anthracycline-induced oxidative stress [20]. Studies have concluded, on the one hand, that trastuzumab-related cardiotoxicity intervened in around 15–20% of patients, where heart failure incidence was under 5%. On the other hand, it has been shown that patients who received trastuzumab had increased levels of cardiac troponin in their blood and that it was associated with a higher risk of irreversible cardiac function [21,22,23]. Nevertheless, other HER2-directed therapies, such as pertuzumab, trastuzumab-emtansine, and lapatinib, are related to a lower risk of cardiac toxicity than trastuzumab. Studies have shown that combined therapy with trastuzumab and pertuzumab did not increase the risk of cardiotoxicity as compared with trastuzumab in monotherapy [24,25,26].

Tyrosine-kinase inhibitors (TKIs) have a major role in cancer treatments and they represent the second most important group of targeted therapies. TKIs impair the addition of a phosphate group to a tyrosine residue within a specific protein. This transfer is actually the main factor in cellular signaling that mediates the cellular functions, survival, and proliferation [27]. Imatinib is a recognized example of TKIs that intervenes with the structure and function of BCR-ABL1 fusion protein. BCR-ABL1 is actually the molecular fingerprint of the Philadelphia chromosome in hematological malignancies, as in the case of chronic myeloid leukemia [28]. The mechanism of toxic action of imatinib upon cardiomyocytes is described as follows: The activation of endoplasmic reticulum stress response, which is continued with the dysfunction of the membrane potential of mitochondria, cytochrome C releasement within the cytosol, and limitation of the ATP cellular level, that finally concludes with cardiomyocyte death [29]. Studies have shown that cardiomyopathy and heart failure produced by imatinib therapy had an incidence under 1% [30,31]. Erlotibin, an inhibitor of epidermal growth factor receptor (EGFR) tyrosine kinase, is recognized as a safer kinase inhibitor in terms of cardiac toxicity. In vivo studies have shown that this reduced risk was attributed to the activation of STAT3 signaling in the heart of mice, as the simultaneous administration of STAT3 inhibitor and erlotinib diminished cardiomyocyte fatty acid oxidation and contractility of the heart, associated with a low risk of cardiotoxicity. Erlotinib cardio-related effects have also been compared with sunitinib and sorafenib that have been recognized for their cardiotoxic adverse effects. The initial supposition upon this difference in adverse effects was associated with the degree of selectivity of the TKIs, where erlotinib was more targeted than the other two compounds. However, it has been demonstrated that the adverse effects on the cardiac system were not related to the selectivity of the treatment, where erlotinib produced more dramatic changes in the kinome and transcriptome of cells [32]. Paradoxically, STAT3 activation in tumors is actually a mechanism of treatment resistance to EGFR inhibitors, for example, pancreatic cancer [33] and non-small cell lung cancer [34,35]. According to this, it has been proposed (and also demonstrated) that the inclusion of a STAT3 inhibitor to a EGFR targeted therapy could enhance the therapeutic potential of the EGFR inhibitor [36,37]. However, taking into account that the specific STAT3 activation is among the cardioprotective signaling element of therapies such erlotinib, these forms of treatments should be closely monitored by a cardiologist.

Guidelines recommend that before administering any potentially cardiotoxic therapeutic, patients must be evaluated by a cardiologist for cardiac function by conducting a 12 lead ECG, followed by a three-dimensional (3D) echocardiography examination or at least a two-dimensional (2D) echocardiography, global longitudinal strain (GLS), and cTn measurement.

**Figure 1:**
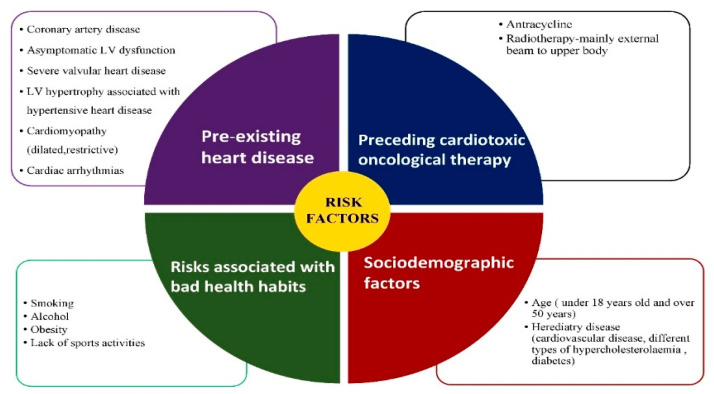
Risk factors that can induce cardiomyopathies.

All these investigations are helpful for determining any potential cardiovascular disease, risk factors, and optimal control of any of the cardiovascular modifications (as shown in Figure 1) [38].

Studies have shown that cardiovascular events such as heart failure and arrhythmias were determined by repeated measurement of cTn levels at one-month follow-up of patients receiving anthracycline treatment. Cardiac dysfunction is defined as a 15% change of global longitudinal strain and 10% less of left ventricular ejection fraction from baseline of the treatment evaluation to < 53% per total [38,39].

There are a considerable number of β-blockers, diuretics, and statins, which can have a protective role against anthracycline-related cardiomyopathy such as carvedilol and nebivolol, spironolactone, and statins [40]. Scientists have been developing a new generation of drugs that includes erythropoietin, which will influence the progenitor cell pool. Two trials on bisoprolol with perindopril and another one with candesartan are ongoing for trastuzumab-induced cardiomyopathy and they have not yet meet their primary end points [41,42,43,44,45].

## 3. Secondary Cardiomyopathies

Type II cardiomyopathy evaluates other factors than a direct toxic effect on cardiomyocytes, which influences the turn down in cardiac function. Knowing these causes can shift the balance in favor of patients’ algorithms for diagnosis and treatment.

Studies have shown that cardiotoxicity was met in up to 30% of the patients who were following schemes with 5-fluorouracil (5-FU) and capecitabine, as shown in Table 1 [46]. The mechanisms of cardiotoxicity are not yet completely understood; however there are studies suggesting that vasoconstriction in coronary microcirculation during 5-FU and capecitabine cures are related to the cardiotoxic events, especially in patients with Takotsubo syndrome [47,48,49]. Other patients can develop vasospasm-related myocardial infarction through the influence of oxidative stress and metabolic disorders in cardiac muscle cells [50]. 5-FU-related cardiotoxicity can be decreased with the prodrug tegafur in combination with uracil which mediates the delivery of 5-fluoropyrimidine, while intercepting with the generation of toxic metabolites [51].

Targeted therapies, such as bevacizumab, have a low risk of cardiac toxicity as compared with 5-FU or with anthracyclines [52]. It has been discovered that heart conditions, such as coronary artery disease (CAD) and hypertension (HTA), which are two main risk factors for cardiomyopathy induced by VEGF inhibitors, had the same mechanism as inhibition of the VEGF pathway, underlining the importance of CAD and HTA as main risk factors [52]. Insulin receptor pathway and platelet-derived growth factor subunit-β pathway are two main pathways that influence sunitinb-related cardiotoxicity. The major role of sunitinib on the heart is to reduce contractility of the heart by reducing the coronary flow reserve [53,54].

Immunotherapies are drugs that are qualified to aim at specific immune cells with the intention of killing malignant cells. One type of cancer immunotherapy, known as chimeric antigen receptor T cell (CAR-T cell) therapy has the role of modulating T cell in recognition of unique signature surface antigens on cancer cells [55]. The first generation of CAR-T cell therapy was associated with a high risk of cardiotoxicity with severe symptoms such as acute respiratory failure, shock, and, in the end, cardiac arrest within 12 h of evolution of the condition. At the autopsy, systemic hemorrhagic microangiopathy and multiorgan ischemia was discovered in the patients [56].

Cytokine release syndrome (CRS) is a condition characterized by tachycardia (in mild forms of CRS), hypotension, arrhythmias, and, in severe form of the condition, reduced left ventricular ejection fraction (LVEF) [57]. In sepsis, there are two major cytokines responsible for LVEF decrease, known as tumor necrosis factor (TNF) and IL-1β and the result of LVEF drop is cardiac remodeling with ventricular dilatation [58]. To prevent this, studies have shown that usage of β-blockers and dobutamine in patients with septic shock improved LVEF [59].

It has been discovered that a second type of T cell-directed immunotherapy, known as bispecific T cell engager therapy (BiTE therapy), could influence heart structure as the first form of therapy, but in a milder version [60]. Other types of immunotherapies include one known as immune checkpoint inhibitors (ICIs). These drugs can influence the heart functions and produce severe conditions such as myocarditis, Takotsubo syndrome, and global cardiomyopathies. ICIs are drugs that aim at T cell inhibition pathways, such as the cytotoxic T lymphocyte antigen 4 (CTLA 4) and programmed cell death 1 (PD1) pathways [61,62,63].

Radiation therapy is one of the most important links in the chain of cancer therapy. The mechanism of this treatment is based on DNA damage that leads to cell aging and cell death. Myocardial fibrosis is produced by infiltration of the leukocytes in the microvascular endothelium of the coronary arteries, which ultimately leads to ischemia and cardiomyocyte necrosis with fibrosis replacement [64,65,66,67].

In type II cardiomyopathy, it is essential to recognize and prevent risk factors. Guidelines recommend reducing cancer therapies that have a major role in increasing the chance of cardiotoxicity. Specifically, the administration of these drugs should be decreased until cardiac function is recovered or until associated factors are controlled. Regarding 5-FU-induced or capecitabine-induced cardiotoxicity, vasodilator drugs are the key treatments, such as nitrates and calcium-channel blockers [68,69]. Scientists have discovered that patients with renal insufficiency and older than 55 years were at higher risk for developing 5-FU-induced cardiotoxicity and this drug should be administrated with caution [68]. Vasodilator therapies should include nitrates and calcium-channel blockers (such as diltiazem). Clinicians should investigate the cardiac rhythm by continuous ECG monitoring. In addition, measurement of BNP (brain-natriuretic peptide) levels and echocardiography might be useful in this group of subjects. In VEGF-inhibitor therapy, proper management of hypertension is a key assessment for this group of patients. One of the main studies in the area, entitled SPRINT, recommends a target of 130/80 mmHg or below for this group of subjects [70,71]. Patients with severe decompensation such as CRS grade III or higher who are following CAR-T cell therapy, should receive prednisone, an anti-inflammatory glucocorticoid and for patients in shock, vasopressors and hemodynamic support is recommended [72]. Patients following radiation therapy are unprotected against harmful radiations and the first measure that is necessary is a reduction in the exposure dose. Experimental studies have shown that a combination of statin and angiotensin-converting enzyme had positive results and should be taken into consideration in this group of patients, but there have been no clinical results so far. The European Society of Cardiovascular Imaging and the Society for Cardiovascular Angiography and Interventions are developing new algorithms for preventing the cardiac consequences of radiation therapy [73,74].

## 4. Type III Cardiomyopathies

Type III cardiomyopathies are described in multiple forms of cancer treatments such as conventional chemotherapies, targeted cancer therapies, and immunotherapies and can lead to various forms of cardiac diseases. Cyclophosphamide is one of the classic examples of conventional chemotherapy that can lead to hemorrhagic myocarditis if the dose is higher than 270 mg/kg for one to four days of usage and also can induce acute heart failure if the dose is over 150 mg/kg [75,76]. This drug can induce various forms of cardiac injury from hemorrhage and thrombosis, to tachyarrhythmia, to pericardial effusion and tamponade. Studies have shown that 2 to 17% of patients treated with cyclophosphamide-based therapy died from myocarditis [76]. Sorafenib is the only TKI associated with fulminant acute myocarditis with cardiogenic shock and the only TKI which has the most atrocious result [77]. The mechanism of action of sorafenib is described by blocking the actions of VEGFR which stops angiogenesis and vasodilator processes, leading to an increase in vascular resistance, hypertension, compensatory hypertrophy, and finally heart failure [78].

Immunotherapies are the most spectacular forms of treatment, with high expectations in cancer therapies, but with fewer studies related to adverse effects than the consecrated drugs. So far, it is known that CTLA4 inhibition has fewer immune-related adverse effects than a combination of CTLA4 and PDL1 [79]. In the literature, conditions such as colitis, dermatitis, and pneumonitis have an incidence of 10% or higher. Most recent studies have shown that myocarditis had the highest mortality among patients (up to 40%) [80]. As in type II cardiotoxicity, immunotherapies affect the heart by decompensation of heart failure, cardiogenic shock, and sudden cardiac death. Pathological studies have shown that ICIs produced structural modifications of the heart with myocardial edema and apical ballooning, heart conditions that have been described in 33% of the patients and in 14% of the patients, respectively [81]. Immunotherapies-related cardiotoxicity has been described multiple ways but has mainly been evaluated through measurement of amino-terminal pro-BNP levels, troponin levels, ECG changes, and echocardiography evaluation. Regarding the circulatory biomarkers, BNP and amino-terminal pro-BNP levels are more superior in detection of cardiomyopathies than troponin levels. The LVEF remains almost normal despite installation of fulminant myocarditis in patients using immunotherapies [81,82]. Proper guidance and prevention are two essential steps in the management of immunotherapies-related cardiotoxicity. The management of severe or fulminant myocarditis includes supportive care, such as inotropic therapy, mechanical circulatory support (or extracorporeal membrane oxygenation) [83,84], as well as 12-lead ECG is recommended. This investigation can detect a decline in the R-wave amplitude which is suggestive of pericardial effusion and reduced myocardial mass. In addition, ECG can detect PR prolongation, heart block, bradycardia, ventricular ectopy, and ventricular tachycardia. Coronary angiography and cardiac positron emission tomography are recommended as a secondary evaluation of the heart, but they are not frequently used, especially because of the cost and their time-consuming character as compared with echocardiography which is cheaper, easier, and faster [85,86]. Immunosuppressive therapies are associated with all forms of myocarditis. Drugs such as immunoglobulin, antithymocyte globulin, infliximab, mycophelonate mofenil, and tacrolimus have shown positive results regarding an effective life-threatening treatment against all forms of myocarditis [87]. New studies have shown that plasmapheresis was more efficient than drugs mentioned earlier, because ICIs have a half-live greater than other chemotherapeutics known until now, for example, 14.5 days for ipilimumab, 25.0 days for pembrolizumab, 26.7 days for nivolumab, and 27.0 days for atezolizumab [88].

## 5. Arrhythmias Related to Neoplastic Treatment

As previously mentioned, there are numerous cardiac conditions caused by cancer therapies, but the most important among them are arrhythmias. As shown in Table 1, the grade of risk is variable between subgroups of cancer treatments. In general, when we refer to arrhythmias, there are two major subgroups differenciated by heartbeats, bradycardia, and tachycardia (with atrial fibrillation, as the most representative pathology).

### 5.1. Bradycardia

The first bradycardia case in connection to cancer treatment was related to paclitaxel, a conventional chemotherapeutic drug, where almost 30% of the patients receiving this form of therapy had bradycardia-induced episodes, but most of them were asymptomatic [89]. Thalidomide is another classic drug that produces bradycardia in almost 50% of the patients treated for myeloma [90].

Targeted cancer therapies are another group of cancer therapeutics that can induce bradycardia, especially sinus bradycardia. Pazopanib is the main representative drug of this group with a chance of 2–19% of inducing bradycardia, but there are also other cancer therapies that produce a low pulse such as sunitinib, crizotinib, and ceritinib [91]. Ibrutinib is another TKI but with more devastating consequences on the heart, such as atrial sinoatrial arrest and asystole, followed by death [92]. 

ICIs can produce different forms of bradycardia, up to atrioventricular block grade III, which is a pacemaker implantation condition [82,93]. In addition, patients receiving ICI therapy can manifest conduction diseases, which evolve with death in almost 50% of the cases [94].

Nevertheless, bradycardia cases have been reported in patients who were on different schemes of radiation therapy and the dose of radiation used could produce fibrosis in the whole heart, from the AV nodal area up to the bundle branches [95].

Regarding the management and treatment of chemotherapy-induced bradycardia, it is recommended that sustained drug dosages be reduced or complementary medications such as β-blockers, calcium-channel blockers be avoided. Nevertheless, K^+^ levels, renal and thyroid function should be rigorously investigated periodically.

### 5.2. Atrial Fibrillation in Cancer Therapies

Atrial fibrillation (AF) is another pathology that can influence treatment in many forms of cancers and can indirectly detect bleeding events in a tumor by anticoagulant usage against AF. For example, cancer antigen CA125 is used as a predictor of AF in postmenopausal women, as well as a tumoral marker for ovarian cancer [96,97]. Symptoms of AF include palpitations, chest discomfort, and dyspnea. This heart condition can influence the prognostic of neoplastic patients by evolving into thromboembolism, myocardial ischemia, and heart failure, conditions that can lead to premature death.

AF is often met in multiple classic chemotherapies, especially in paclitaxel and melphalan. Eight per cent of patients receiving melphalan have developed AF, and studies have shown that left atrial enlargement was the main risk factor for developing such conditions [98,99].

AF has been reported in patients receiving TKIs, especially ibrutinib, where it has been shown that up to 16% of the patients developed the condition [100,101].

Studies have shown that patients with cancer and AF had high levels of C-reactive protein (CRP) in their blood. In addition, patients receiving CAR-T cell therapy have a higher risk of developing AF than patients receiving other ICIs, and, importantly, AF was also present in young patients receiving CAR-T cell therapy [102].

AF is responsible for cardiac conditions such as pericarditis or cardiomyopathies in oncological patients. Management and prevention of AF in oncological patients have the same goals as in the general population, with β-blockers such as atenolol and metoprolol used as first-line treatment agents. In addition, Ca^2+^ channel blockers and digoxin are recommended for reduction in the heart rate, but it is known that ibrutinib can increase the plasma levels of carvedilol, verapamil, diltiazem, amiodarone, and digoxin, and therefore caution is recommended when these drugs are used in combination for AF management [103].

Anticoagulants have a benefical and necessary role in AF, but it must be administered with precautions in patients receiving cancer therapies. Ibrutinib, for example, is associated with a major risk of bleeding in up to 7% of the patients, by inhibiting the von Willebrand factor and collagen-mediated platelet activation [104,105]. A combination of antiplatelet agents and anticoagulation therapy multiplies the risk of bleeding both in the general population and in the oncological population as well. On the basis of the CHA_2_DS_2_-VASc score and HAS-BLED score, low-molecular-weight heparin or direct oral anticoagulants such as NOACs are recommended by both cardiologist and oncologist to prevent complications mentioned before in neoplastic patients [106,107,108,109].

## 6. Complementary Investigations for Diagnosing Cardiotoxicity

**Figure 2:**
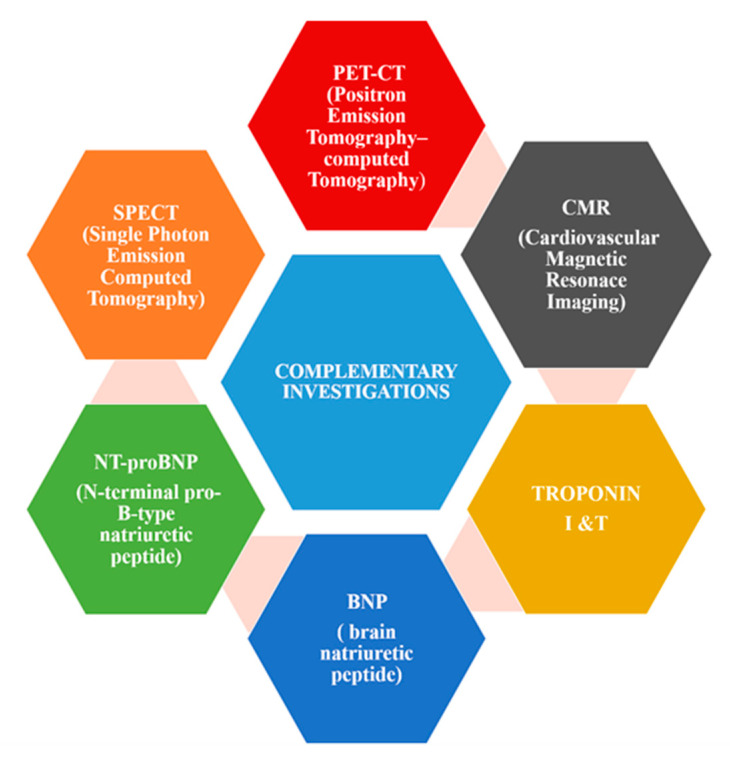
Complementary investigations for diagnosing cardiotoxicity.

On a daily basis, it is recommended that the clinical team in oncology should include a cardiologist, in addition to the oncologists, for early detection of cardiovascular diseases before the oncological cure is set in motion, with the goal of recommending optimal treatments for neoplastic patients. Echocardiography is the gold standard and the most used imaging technique for the evaluation of structural and functional abnormalities of the heart. Other investigations include CMR, biomarkers for myocardial injury, SPECT, and PET imaging (Figure 2).

Cardiovascular magnetic resonance imaging (CMR) is used to evaluate heart dilatation with high precession. The major role of CMR is to demonstrate a non-ischemic pattern of late gadolinium enhancement (LGE) most consistent with a myocarditis. Troponin I is a more specific subunit than troponin T and it remains detectable longer than troponin T (10–14 days versus 4–7 days). Troponin subunit levels are both used to evaluate the presence of myocardial injury [110]. BNP (brain natriuretic peptide) and its subunit NT-proBNP (N-terminal pro-B-type natriuretic peptide) are used to evaluate heart failure and myocardial dysfunction in patients using cancer therapies and they are effective predictors of short- and long-term major adverse events after ischemic heart disease [111,112]. Single photon emission computed tomography (SPECT) imaging of NSCLC (non-small cell lung carcinoma) with a 99mTc-labeled sdAb (99mTc-NM-01) that binds in a specific manner to human PD-L1 is feasible to use in correlation with PD-L1 immunohistochemistry results. This method is a complementary investigation to evaluate the myocardial perfusion of the heart.

Positron emission tomography/computed tomography (better known as PET-CT or PET/CT) is a technique of the nuclear medicine spectrum which brings together, in a single gantry, a positron emission tomography (PET) scanner and an X-ray computed tomography (CT) scanner, to gather sequential images from both scanners in the same analysis, which are combined into a single superposed (co-registered) image. Thus, PET imaging provides key information regarding the spatial distribution of metabolic or biochemical activity in cancer.

## 7. Gender and Age-Related Differences in Chemotherapy-Induced Cardiotoxicity

Cancer therapies must be adjusted differentially for pediatric patients versus elder patients (older than 65 years old) [113] because they respond different to the oncological treatment. For example, breast cancer biology may differ in older patients who receive less intensive treatment and who have a higher risk of mortality. In this group, tolerance of treatment, as well as life expectancy may be significantly reduced, are factors that should be considered when new oncological treatment is recommended [113,114,115]. Among pediatric patients, studies have shown that cancer therapies had more positive results. Any cancer types that are discovered at an early age are associated with higher life expectancy and also a higher tolerance to treatment. Studies have discovered that pediatric patients have a superior immunity as compared with seniors, because people over 65 years of age develop different types of diseases (such as diabetes mellitus) which can influence and unbalance the immune system before any cancer is discovered in this group [116,117]. Moreover, the risk of developing cardiac toxicities is more common in elderly patients due to an already susceptible phenotype characterized by the presence of chronic heart disease.

There are also differences between genders in terms of cancer evolution and how they respond to treatment. Cardiovascular cells contain functional estrogen (ER) and androgen (AR) receptors and are targets for sex hormone action, which can influence many physiological and pathological processes, including vascular and myocardial cell homeostasis. Two ERs, ERα and ERβ, have been described. 17β-estradiol (E2) may have genomic and non-genomic effects. The genomic effects involve binding of hormones on hormone responsive elements and further regulation of cardiac specific gene expression [118]. Non-genomic effects involve rapid, within seconds or minutes, signaling effects through activation of non-nuclear membrane-associated ERs [119]. The relative importance of genomic and non-genomic effects of ERα and ERβ in the cardiomyocyte is still a matter of debate [120,121,122]. In humans, beyond these biological aspects, differences in lifestyle between women and men such as smoking status, alcohol consumption, or dietary habits could also partly explain this sexually dimorphic gene expression, habits which are known to be associated with the incidence of heart failure [123].

## 8. Conclusions

The new era of oncology emerges with new treatment options for malignant diseases that ultimately lead to low rates of tumor progression and a significant increase in favorable prognostics. However, one of the most important aspects in terms of obtaining complete clinical success is represented by the evaluation of secondary conditions and proper management of the entire clinical profile of the patients, especially cardiologic evaluation.

Cancer therapy has been evolving rapidly on the uprising hill of developing new drugs, from classic therapies to targeted molecular therapies and especially, with higher expectations and immunotherapies. Due to the novelty of certain therapies, further studies are needed to consolidate cardiovascular imaging and new biomarkers to decide when to intervene and stop a potentially cardiotoxic therapy. Together we can develop a better tomorrow!

## Figures and Tables

**Table 1:** Therapies that can induce arrythmias.

	Chemotherapeutic Agent	Risk for Arrythmia
ALK inhibitors *	**Doxorubicin**	**high risk**
	**Epirubicin**	**high risk**
	**Idarubicin**	**high risk**
	**Mitoxantrone**	**high risk**
**Alkylating agents**	**Busulfan**	**high risk**
	**Cyclophosphamide A**	**low risk**
	**Ifosfamide**	**no risk**
	**Melphalan**	**medium risk**
CDK4/CDK6 inhibitors **	**Ribociclib**	**medium risk**
**Immune checkpoint inhibitors**	**Ipilimumab (anti-CTLA4)**	**low risk**
	**Nivolumab (anti-PD1)**	**low risk**
	**Pembrolizumab (anti-PD1)**	**low risk**
**Microtubule-binding agents**	**Docetaxel**	**medium risk**
	**Paclitaxel**	**low risk**
	**Vinblastine**	**no risk**
	**Vincristine**	**no risk**

* ALK (anaplastic lymphoma kinase) inhibitors; ** CDK4/CDK6 (cyclin-dependent kinase 4 and 6) inhibitors.

## References

[1] Weir H.K., Anderson R.N., King C.S.M., Soman A., Thompson T.D., Hong Y., Moller B., Leadbetter S. (2016). Heart Disease and Cancer Deaths—Trends and Projections in the United States, 1969–2020. Prev. Chronic Dis..

[2] American Cancer Society (2017). Cancer Facts and Figures 2017. Genes Dev..

[3] Tan C., Tasaka H., Yu K.-P., Murphy M.L., Karnofsky D.A. (1967). Daunomycin, an antitumor antibiotic, in the treatment of neoplastic disease. Clinical evaluation with special reference to childhood leukemia. Cancer.

[4] Rowinsky E.K., McGuire W.P., Guarnieri T., Fisherman J.S., Christian M.C., Donehower R.C. (1991). Cardiac disturbances during the administration of taxol. J. Clin. Oncol..

[5] Sorrentino M.F., Kim J., Foderaro A.E., Truesdell A.G. (2012). 5-fluorouracil induced cardiotoxicity: Review of the literature. Cardiol. J..

[6] Herrmann J. (2020). Adverse cardiac effects of cancer therapies: Cardiotoxicity and arrhythmia. Nat. Rev. Cardiol..

[7] Gianni L., Herman E.H., Lipshultz S.E., Minotti G., Sarvazyan N., Sawyer D.B. (2008). Anthracycline cardiotoxicity: From bench to bedside. J. Clin. Oncol..

[8] Peng X., Chen B., Lim C.C., Sawyer D.B. (2005). The cardiotoxicology of anthracycline chemotherapeutics: Translating molecular mechanism into preventative medicine. Mol. Interv..

[9] Unverferth D.V., Magorien R.D., Unverferth B.P., Talley R.L., Balcerzak S.P., Baba N. (1981). Human myocardial morphologic and functional changes in the first 24 h after doxorubicin administration. Cancer Treat. Rep..

[10] Herrmann J., Lerman A., Sandhu N.P., Villarraga H.R., Mulvagh S.L., Kohli M. (2014). Evaluation and management of patients with heart disease and cancer: Cardio-oncology. Mayo Clin. Proc..

[11] Schafer F.Q., Buettner G.R. (2001). Redox environment of the cell as viewed through the redox state of the glutathione disulfide/glutathione couple. Free Radic. Biol. Med..

[12] Merlot A.M., Kalinowski D.S., Richardson D.R. (2013). Novel chelators for cancer treatment: Where are we now?. Antioxid. Redox Signal..

[13] Bristow M.R., Thompson P.D., Martin R.P., Mason J.W., Billingham M.E., Harrison D.C. (1978). Early anthracycline cardiotoxicity. Am. J. Med..

[14] Bristow M.R., Billingham M.E., Mason J.W., Daniels J.R. (1978). Clinical spectrum of anthracycline antibiotic cardiotoxicity. Cancer Treat. Rep..

[15] Carter P., Presta L., Gorman C.M., Ridgway J.B.B., Henner D., Wong W.L.T., Rowland A.M., Kotts C., Carver M.E., Shepard H.M. (1992). Humanization of an anti-p185HER2 antibody for human cancer therapy. Proc. Natl. Acad. Sci. USA.

[16] Moasser M.M. (2007). The oncogene HER2: Its signaling and transforming functions and its role in human cancer pathogenesis. Oncogene.

[17] Moasser M.M., Krop I.E. (2015). The evolving landscape of HER2 targeting in breast cancer. JAMA Oncol..

[18] Slamon D.J., Leyland-Jones B., Shak S., Fuchs H., Paton V., Bajamonde A., Fleming T., Eiermann W., Wolter J., Pegram M. (2001). Use of chemotherapy plus a monoclonal antibody against her2 for metastatic breast cancer that overexpresses HER2. N. Engl. J. Med..

[19] Bowles E.J.A., Wellman R., Feigelson H.S., Onitilo A.A., Freedman A.N., Delate T., Allen L.A., Nekhlyudov L., Goddard K.A.B., Davis R.L. (2012). Risk of heart failure in breast cancer patients after anthracycline and trastuzumab treatment: A retrospective cohort study. J. Natl. Cancer Inst..

[20] Sawyer D.B., Zuppinger C., Miller T.A., Eppenberger H.M., Suter T.M. (2002). Modulation of anthracycline-induced myofibrillar disarray in rat ventricular myocytes by neuregulin-1β and anti-erbB2: Potential mechanism for trastuzumab-induced cardiotoxicity. Circulation.

[21] Farolfi A., Melegari E., Aquilina M., Scarpi E., Ibrahim T., Maltoni R., Sarti S., Cecconetto L., Pietri E., Ferrario C. (2013). Trastuzumab-induced cardiotoxicity in early breast cancer patients: A retrospective study of possible risk and protective factors. Heart.

[22] Nowsheen S., Aziz K., Park J.Y., Lerman A., Villarraga H.R., Ruddy K.J., Herrmann J. (2018). Trastuzumab in female breast cancer patients with reduced left ventricular ejection fraction. J. Am. Heart Assoc..

[23] Cardinale D., Colombo A., Torrisi R., Sandri M.T., Civelli M., Salvatici M., Lamantia G., Colombo N., Cortinovis S., Dessanai M.A. (2010). Trastuzumab-induced cardiotoxicity: Clinical and prognostic implications of troponin I evaluation. J. Clin. Oncol..

[24] Baselga J., Cortés J., Kim S.B., Im S.A., Hegg R., Im Y.H., Roman L., Pedrini J.L., Pienkowski T., Knott A. (2012). Pertuzumab plus trastuzumab plus docetaxel for metastatic breast cancer. N. Engl. J. Med..

[25] Swain S.M., Baselga J., Kim S.B., Ro J., Semiglazov V., Campone M., Ciruelos E., Ferrero J.M., Schneeweiss A., Heeson S. (2014). Pertuzumab, trastuzumab, and docetaxel in HER2-positive metastatic breast cancer. N. Engl. J. Med..

[26] Piccart-Gebhart M., Holmes E., Baselga J., De Azambuja E., Dueck A.C., Viale G., Zujewski J.A., Goldhirsch A., Armour A., Pritchard K.I. (2016). Adjuvant lapatinib and trastuzumab for early human epidermal growth factor receptor 2-positive breast cancer: Results From the randomized phase III adjuvant lapatinib and/or trastuzumab treatment optimization trial. J. Clin. Oncol..

[27] Bhullar K.S., Lagarón N.O., McGowan E.M., Parmar I., Jha A., Hubbard B.P., Rupasinghe H.P.V. (2018). Kinase-targeted cancer therapies: Progress, challenges and future directions. Mol. Cancer.

[28] Thompson P.A., Kantarjian H.M., Cortes J.E. (2015). Diagnosis and Treatment of Chronic Myeloid Leukemia in 2015. Mayo Clin. Proc..

[29] Kerkelä R., Grazette L., Yacobi R., Iliescu C., Patten R., Beahm C., Walters B., Shevtsov S., Pesant S., Clubb F.J. (2006). Cardiotoxicity of the cancer therapeutic agent imatinib mesylate. Nat. Med..

[30] Ribeiro A.L., Marcolino M.S., Bittencourt H.N.S., Barbosa M.M., Nunes M.D.C.P., Xavier V.F., Clementino N.C.D. (2008). An evaluation of the cardiotoxicity of imatinib mesylate. Leuk. Res..

[31] Estabragh Z.R., Knight K., Watmough S.J., Lane S., Vinjamuri S., Hart G., Clark R.E. (2011). A prospective evaluation of cardiac function in patients with chronic myeloid leukaemia treated with imatinib. Leuk. Res..

[32] Stuhlmiller T.J., Zawistowski J.S., Chen X., Sciaky N., Angus S.P., Hicks S.T., Parry T.L., Huang W., Beak J.Y., Willis M.S. (2017). Kinome and transcriptome profiling reveal broad and distinct activities of erlotinib, sunitinib, and sorafenib in the mouse heart and suggest cardiotoxicity from combined signal transducer and activator of transcription and epidermal growth factor recepto. J. Am. Heart Assoc..

[33] Burris H.A., Moore M.J., Andersen J., Green M.R., Rothenberg M.L., Modiano M.R., Cripps M.C., Portenoy R.K., Storniolo A.M., Tarassoff P. (1997). Improvements in survival and clinical benefit with gemcitabine as first- line therapy for patients with advanced pancreas cancer: A randomized trial. J. Clin. Oncol..

[34] Yeh H.H., Lai W.W., Chen H.H.W., Liu H.S., Su W.C. (2006). Autocrine IL-6-induced Stat3 activation contributes to the pathogenesis of lung adenocarcinoma and malignant pleural effusion. Oncogene.

[35] Gao S.P., Mark K.G., Leslie K., Pao W., Motoi N., Gerald W.L., Travis W.D., Bornmann W., Veach D., Clarkson B. (2007). Mutations in the EGFR kinase domain mediate STAT3 activation via IL-6 production in human lung adenocarcinomas. J. Clin. Investig..

[36] Glaysher S., Bolton L.M., Johnson P., Atkey N., Dyson M., Torrance C., Cree I.A. (2013). Targeting EGFR and PI3K pathways in ovarian cancer. Br. J. Cancer.

[37] Lo H.W., Cao X., Zhu H., Ali-Osman F. (2008). Constitutively activated STAT3 frequently coexpresses with epidermal growth factor receptor in high-grade gliomas and targeting STAT3 sensitizes them to iressa and alkylators. Clin. Cancer Res..

[38] Plana J.C., Galderisi M., Barac A., Ewer M.S., Ky B., Scherrer-Crosbie M., Ganame J., Sebag I.A., Agler D.A., Badano L.P. (2014). Expert consensus for multimodality imaging evaluation of adult patients during and after cancer therapy: A report from the American Society of Echocardiography and the European Association of Cardiovascular Imaging. Eur. Heart J. Cardiovasc. Imaging.

[39] Cardinale D., Sandri M.T., Colombo A., Colombo N., Boeri M., Lamantia G., Civelli M., Peccatori F., Martinelli G., Fiorentini C. (2004). Prognostic value of troponin I in cardiac risk stratification of cancer patients undergoing high-dose chemotherapy. Circulation.

[40] Avila M.S., Ayub-Ferreira S.M., Wanderley D.B.M.R., Cruz D.D.F., Brandão G.S.M., Rigaud V.O.C., Higuchi-dos-Santos M.H., Hajjar L.A., Filho R.K., Hoff P.M. (2018). Carvedilol for Prevention of Chemotherapy-Related Cardiotoxicity: The CECCY Trial. J. Am. Coll. Cardiol..

[41] Urbanek K., Frati C., Graiani G., Madeddu D., Falco A., Cavalli S., Lorusso B., Gervasi A., Prezioso L., Savi M. (2015). Cardioprotection by Targeting the Pool of Resident and Extracardiac Progenitors. Curr. Drug Targets.

[42] Hamed S., Barshack I., Luboshits G., Wexler D., Deutsch V., Keren G., George J. (2006). Erythropoietin improves myocardial performance in doxorubicin-induced cardiomyopathy. Eur. Heart J..

[43] Hoch M., Fischer P., Stapel B., Missol-Kolka E., Sekkali B., Scherr M., Favret F., Braun T., Eder M., Schuster-Gossler K. (2011). Erythropoietin preserves the endothelial differentiation capacity of cardiac progenitor cells and reduces heart failure during anticancer therapies. Cell Stem Cell.

[44] Pituskin E., Mackey J.R., Koshman S., Jassal D., Pitz M., Haykowsky M.J., Pagano J.J., Chow K., Thompson R.B., Vos L.J. (2017). Multidisciplinary approach to novel therapies in cardio-oncology research (MANTICORE 101-Breast): A randomized trial for the prevention of trastuzumab-associated cardiotoxicity. J. Clin. Oncol..

[45] Boekhout A.H., Gietema J.A., Kerklaan B.M., VanWerkhoven E.D., Altena R., Honkoop A., Los M., Smit W.M., Nieboer P., Smorenburg C.H. (2016). Angiotensin II Receptor inhibition with candesartan to prevent trastuzumab-related cardiotoxic effects in patients with early breast cancer a randomized clinical trial. JAMA Oncol..

[46] Cerny J., Hassan A., Smith C., Piperdi B. (2009). Coronary vasospasm with myocardial stunning in a patient with colon cancer receiving adjuvant chemotherapy with FOLFOX regimen. Clin. Colorectal Cancer.

[47] Basselin C., Fontanges T., Descotes J., Chevalier P., Bui-Xuan B., Feinard G., Timour Q. (2011). 5-Fluorouracil-induced Tako-Tsubo-like syndrome. Pharmacotherapy.

[48] Gianni M., Dentali F., Lonn E. (2009). 5 flourouracil-induced apical ballooning syndrome: A case report. Blood Coagul. Fibrinolysis.

[49] Grunwald M.R., Howie L., Diaz L.A. (2012). Takotsubo cardiomyopathy and fluorouracil: Case report and review of the literature. J. Clin. Oncol..

[50] Tsibiribi P., Bui-Xuan C., Bui-Xuan B., Lombard-Bohas C., Duperret S., Belkhiria M., Tabib A., Maujean G., Descotes J., Timour Q. (2006). Cardiac lesions induced by 5-fluorouracil in the rabbit. Hum. Exp. Toxicol..

[51] Papanastasopoulos P., Stebbing J. (2014). Molecular basis of 5-fluorouracil-related toxicity: Lessons from clinical practice. Anticancer Res..

[52] Touyz R.M., Herrmann J. (2018). Cardiotoxicity with vascular endothelial growth factor inhibitor therapy. NPJ Precis. Oncol..

[53] Lazarus A., Keshet E. (2011). Vascular endothelial growth factor and vascular homeostasis. Proc. Am. Thorac. Soc..

[54] Maharaj A.S.R., D’Amore P.A. (2007). Roles for VEGF in the adult. Microvasc. Res..

[55] Brudno J.N., Kochenderfer J.N. (2018). Chimeric antigen receptor T-cell therapies for lymphoma. Nat. Rev. Clin. Oncol..

[56] Morgan R.A., Yang J.C., Kitano M., Dudley M.E., Laurencot C.M., Rosenberg S.A. (2010). Case report of a serious adverse event following the administration of t cells transduced with a chimeric antigen receptor recognizing ERBB2. Mol. Ther..

[57] Neelapu S.S., Tummala S., Kebriaei P., Wierda W., Gutierrez C., Locke F.L., Komanduri K.V., Lin Y., Jain N., Daver N. (2018). Chimeric antigen receptor T-cell therapy-assessment and management of toxicities. Nat. Rev. Clin. Oncol..

[58] Krishnagopalan S., Kumar A., Parrillo J.E., Kumar A. (2002). Myocardial dysfunction in the patient with sepsis. Curr. Opin. Crit. Care.

[59] Morelli A., Ertmer C., Westphal M., Rehberg S., Kampmeier T., Ligges S., Orecchioni A., D’Egidio A., D’Ippoliti F., Raffone C. (2013). Effect of heart rate control with esmolol on hemodynamic and clinical outcomes in patients with septic shock: A randomized clinical trial. JAMA J. Am. Med. Assoc..

[60] Slaney C.Y., Wang P., Darcy P.K., Kershaw M.H. (2018). CARs versus biTEs: A comparison between T cell–redirection strategies for cancer treatment. Cancer Discov..

[61] Pardoll D.M. (2012). The blockade of immune checkpoints in cancer immunotherapy. Nat. Rev. Cancer.

[62] Sury K., Perazella M.A., Shirali A.C. (2018). Cardiorenal complications of immune checkpoint inhibitors. Nat. Rev. Nephrol..

[63] Ederhy S., Cautela J., Ancedy Y., Escudier M., Thuny F., Cohen A. (2018). Takotsubo-Like Syndrome in Cancer Patients Treated With Immune Checkpoint Inhibitors. JACC Cardiovasc. Imaging.

[64] Saiki H., Petersen I.A., Scott C.G., Bailey K.R., Dunlay S.M., Finley R.R., Ruddy K.J., Yan E., Redfield M.M. (2017). Risk of Heart Failure with Preserved Ejection Fraction in Older Women after Contemporary Radiotherapy for Breast Cancer. Circulation.

[65] Heselich A., Frieß J.L., Ritter S., Benz N.P., Layer P.G., Thielemann C. (2018). High LET radiation shows no major cellular and functional effects on primary cardiomyocytes in vitro. Life Sci. Space Res..

[66] Fajardo L.F., Stewart J.R. (1970). Experimental radiation-induced heart disease. I. Light microscopic studies. Am. J. Pathol..

[67] Stewart F.A., Seemann I., Hoving S., Russell N.S. (2013). Understanding radiation-induced cardiovascular damage and strategies for intervention. Clin. Oncol..

[68] Jensen S.A., Sørensen J.B. (2006). Risk factors and prevention of cardiotoxicity induced by 5-fluorouracil or capecitabine. Cancer Chemother. Pharmacol..

[69] Meyer C.C., Calis K.A., Burke L.B., Walawander C.A., Grasela T.H. (1997). Symptomatic cardiotoxicity associated with 5-fluorouracil. Pharmacotherapy.

[70] Truitt R., Mu A., Corbin E.A., Vite A., Brandimarto J., Ky B., Margulies K.B. (2018). Increased Afterload Augments Sunitinib-Induced Cardiotoxicity in an Engineered Cardiac Microtissue Model. JACC Basic Transl. Sci..

[71] Touyz R.M., Herrmann S.M.S., Herrmann J. (2018). Vascular toxicities with VEGF inhibitor therapies–focus on hypertension and arterial thrombotic events. J. Am. Soc. Hypertens..

[72] Annane D., Ouanes-Besbes L., de Backer D., Du B., Gordon A.C., Hernández G., Olsen K.M., Osborn T.M., Peake S., Russell J.A. (2018). A global perspective on vasoactive agents in shock. Intensive Care Med..

[73] Lancellotti P., Nkomo V.T., Badano L.P., Bergler J., Bogaert J., Davin L., Cosyns B., Coucke P., Dulgheru R., Edvardsen T. (2013). Expert Consensus for multi-modality imaging evaluation of cardiovascular complications of radiotherapy in adults: A report from the European association of cardiovascular imaging and the American society of echocardiography. J. Am. Soc. Echocardiogr..

[74] Iliescu C.A., Grines C.L., Herrmann J., Yang E.H., Cilingiroglu M., Charitakis K., Hakeem A., Toutouzas K.P., Leesar M.A., Marmagkiolis K. (2016). SCAI Expert consensus statement: Evaluation, management, and special considerations of cardio-oncology patients in the cardiac catheterization laboratory (endorsed by the cardiological society of India, and sociedad Latino Americana de Cardiologbox drawin. Catheter. Cardiovasc. Interv..

[75] Appelbaum F.R., Strauchen J.A., Graw R.G., Savage D.D., Kent K.M., Ferrans V.J., Herzig G.P. (1976). Acute lethal carditis caused by high-dose combination chemotherapy. A Unique Clinical and Pathological Entity. Lancet.

[76] Dhesi S., Chu M., Blevins G., Paterson I., Larratt L., Oudit G., Kim D. (2013). Cyclophosphamide-Induced Cardiomyopathy: A Case Report, Review, and Recommendations for Management. J. Investig. Med. High Impact Case Rep..

[77] Asawaeer M., Barton D., Radio S., Chatzizisis Y.S. (2018). Tyrosine Kinase Inhibitor-Induced Acute Myocarditis, Myositis, and Cardiogenic Shock. Methodist Debakey Cardiovasc. J..

[78] Syrigos K.N., Karapanagiotou E., Boura P., Manegold C., Harrington K. (2011). Bevacizumab-induced hypertension: Pathogenesis and management. BioDrugs.

[79] Eigentler T.K., Hassel J.C., Berking C., Aberle J., Bachmann O., Grünwald V., Kähler K.C., Loquai C., Reinmuth N., Steins M. (2016). Diagnosis, monitoring and management of immune-related adverse drug reactions of anti-PD-1 antibody therapy. Cancer Treat. Rev..

[80] Lyon A.R., Yousaf N., Battisti N.M.L., Moslehi J., Larkin J. (2018). Immune checkpoint inhibitors and cardiovascular toxicity. Lancet Oncol..

[81] Escudier M., Cautela J., Malissen N., Ancedy Y., Orabona M., Pinto J., Monestier S., Grob J.J., Scemama U., Jacquier A. (2017). Clinical features, management, and outcomes of immune checkpoint inhibitor-related cardiotoxicity. Circulation.

[82] Johnson D.B., Balko J.M., Compton M.L., Chalkias S., Gorham J., Xu Y., Hicks M., Puzanov I., Alexander M.R., Bloomer T.L. (2016). Fulminant myocarditis with combination immune checkpoint blockade. N. Engl. J. Med..

[83] Freilich M., Stub D., Esmore D., Negri J., Salamonsen R., Bergin P., Leet A., Richardson M., Taylor A., Woodard J. (2009). Recovery From Anthracycline Cardiomyopathy After Long-term Support With a Continuous Flow Left Ventricular Assist Device. J. Heart Lung Transpl..

[84] Arangalage D., Delyon J., Lermuzeaux M., Ekpe K., Ederhy S., Pages C., Lebbé C. (2017). Survival after fulminant myocarditis induced by immune-checkpoint inhibitors. Ann. Intern. Med..

[85] O’Regan D.P., Cook S.A. (2011). Myocarditis or myocardial infarction? MRI can help. Heart.

[86] Miller E.J., Culver D.A. (2018). Establishing an Evidence-Based Method to Diagnose Cardiac Sarcoidosis: The Complementary Use of Cardiac Magnetic Resonance Imaging and FDG-PET. Circ. Cardiovasc. Imaging.

[87] Wang D.Y., Okoye G.D., Neilan T.G., Johnson D.B., Moslehi J.J. (2017). Cardiovascular Toxicities Associated with Cancer Immunotherapies. Curr. Cardiol. Rep..

[88] Salem J.E., Allenbach Y., Kerneis M. (2019). Abatacept for severe immune checkpoint inhibitor–associated myocarditis. N. Engl. J. Med..

[89] Arbuck S.G., Strauss H., Rowinsky E., Christian M., Suffness M., Adams J., Oakes M., McGuire W., Reed E., Gibbs H. (1993). A reassessment of cardiac toxicity associated with Taxol. J. Natl. Cancer Inst. Monogr..

[90] Tamargo J., Caballero R., Delpón E. (2015). Cancer Chemotherapy and Cardiac Arrhythmias: A Review. Drug Saf..

[91] Lele A.V., Clutter S., Price E., De Ruyter M.L. (2013). Severe hypothyroidism presenting as myxedema coma in the postoperative period in a patient taking sunitinib: Case report and review of literature. J. Clin. Anesth..

[92] Mathur K., Saini A., Ellenbogen K.A., Shepard R.K. (2017). Profound Sinoatrial Arrest Associated with Ibrutinib. Case Rep. Oncol. Med..

[93] Cooper L.T. (2009). Myocarditis. N. Engl. J. Med..

[94] Mir H., Alhussein M., Alrashidi S., Alzayer H., Alshatti A., Valettas N., Mukherjee S.D., Nair V., Leong D.P. (2018). Cardiac Complications Associated With Checkpoint Inhibition: A Systematic Review of the Literature in an Important Emerging Area. Can. J. Cardiol..

[95] Orzan F., Brusca A., Gaita F., Giustetto C., Figliomeni M.C., Libero L. (1993). Associated cardiac lesions in patients with radiation-induced complete heart block. Int. J. Cardiol..

[96] Conen D., Wong J.A., Sandhu R.K., Cook N.R., Lee I.M., Buring J.E., Albert C.M. (2016). Risk of malignant cancer among women with new-onset atrial fibrillation. JAMA Cardiol..

[97] Sekiguchi H., Shimamoto K., Takano M., Kimura M., Takahashi Y., Tatsumi F., Watanabe E., Jujo K., Ishizuka N., Kawana M. (2017). Cancer antigen-125 plasma level as a biomarker of new-onset atrial fibrillation in postmenopausal women. Heart.

[98] Feliz V., Saiyad S., Ramarao S.M., Khan H., Leonelli F., Guglin M. (2011). Melphalan-induced supraventricular tachycardia: Incidence and risk factors. Clin. Cardiol..

[99] Zhao D., Chen J., Liu X., Long X., Cao L., Wang J. (2018). Atrial fibrillation following treatment with paclitaxel: A case report. Biomed. Rep..

[100] Shanafelt T.D., Parikh S.A., Noseworthy P.A., Goede V., Chaffee K.G., Bahlo J., Call T.G., Schwager S.M., Ding W., Eichhorst B. (2017). Atrial fibrillation in patients with chronic lymphocytic leukemia (CLL). Leuk. Lymphoma.

[101] Leong D.P., Caron F., Hillis C., Duan A., Healey J.S., Fraser G., Siegal D. (2016). The risk of atrial fibrillation with ibrutinib use: A systematic review and meta-analysis. Blood.

[102] Davila M.L., Riviere I., Wang X., Bartido S., Park J., Curran K., Chung S.S., Stefanski J., Borquez-Ojeda O., Olszewska M. (2014). Efficacy and toxicity management of 19-28z CAR T cell therapy in B cell acute lymphoblastic leukemia. Sci. Transl. Med..

[103] Vrontikis A., Carey J., Gilreath J.A., Halwani A., Stephens D.M., Sweetenham J.W. (2016). Proposed algorithm for managing Ibrutinib-related atrial fibrillation. Oncology (US).

[104] Aguilar C. (2018). Ibrutinib-related bleeding: Pathogenesis, clinical implications and management. Blood Coagul. Fibrinolysis.

[105] Busygina K., Jamasbi J., Seiler T., Deckmyn H., Weber C., Brandl R., Lorenz R., Siess W. (2018). Oral Bruton tyrosine kinase inhibitors selectively block atherosclerotic plaque-triggered thrombus formation in humans. Blood.

[106] Shah S., Norby F.L., Datta Y.H., Lutsey P.L., MacLehose R.F., Chen L.Y., Alonso A. (2018). Comparative effectiveness of direct oral anticoagulants and warfarin in patients with cancer and atrial fibrillation. Blood Adv..

[107] Xiang E., Ahuja T., Raco V., Cirrone F., Green D., Papadopoulos J. (2018). Anticoagulation prescribing patterns in patients with cancer. J. Thromb. Thrombolysis.

[108] Lee Y.J., Park J.K., Uhm J.S., Kim J.Y., Pak H.N., Lee M.H., Sung J.H., Joung B. (2016). Bleeding risk and major adverse events in patients with cancer on oral anticoagulation therapy. Int. J. Cardiol..

[109] Friberg L., Skeppholm M., Terént A. (2015). Benefit of anticoagulation unlikely in patients with atrial fibrillation and a CHA2DS2-VASc score of 1. J. Am. Coll. Cardiol..

[110] Morrow D.A., Cannon C.P., Jesse R.L., Newby L.K., Ravkilde J., Storrow A.B., Wu A.H.B., Christenson R.H., Apple F.S., Francis G. (2007). National Academy of Clinical Biochemistry Laboratory Medicine Practice Guidelines: Clinical characteristics and utilization of biochemical markers in acute coronary syndromes. Clin. Chem..

[111] De Lemos J.A., Morrow D.A., Bentley J.H., Omland T., Sabatine M.S., McCabe C.H., Hall C., Cannon C.P., Braunwald E. (2001). The prognostic value of B-type natriuretic peptide in patients with acute coronary syndromes. N. Engl. J. Med..

[112] Richards A.M., Nicholls M.G., Espiner E.A., Lainchbury J.G., Troughton R.W., Elliott J., Frampton C., Turner J., Crozier I.G., Yandle T.G. (2003). B-type natriuretic peptides and ejection fraction for prognosis after myocardial infarction. Circulation.

[113] Biganzoli L., Wildiers H., Oakman C., Marotti L., Loibl S., Kunkler I., Reed M., Ciatto S., Voogd A.C., Brain E. (2012). Management of elderly patients with breast cancer: Updated recommendations of the International Society of Geriatric Oncology (SIOG) and European Society of Breast Cancer Specialists (EUSOMA). Lancet Oncol..

[114] Water W.V.D., Kiderlen M., Bastiaannet E., Siesling S., Westendorp R.G.J., Velde C.J.H.V.D., Nortier J.W.R., Seynaeve C., De Craen A.J.M., Liefers G.J. (2014). External validity of a trial comprised of elderly patients with hormone receptor-positive breast cancer. J. Natl. Cancer Inst..

[115] Water W.V.D., Markopoulos C., Velde C.J.H.V.D., Seynaeve C., Hasenburg A., Rea D., Putter H., Nortier J.W.R., De Craen A.J.M., Hille E.T.M. (2012). Association between age at diagnosis and disease-specific mortality among postmenopausal women with hormone receptor-positive breast cancer. JAMA J. Am. Med. Assoc..

[116] Children’s Oncology Group (2008). Long-Term Follow-Up Guidelines for Survivors of Childhood, Adolescent and Young Adult Cancers.

[117] Kavey R.E.W., Allada V., Daniels S.R., Hayman L.L., McCrindle B.W., Newburger J.W., Parekh R.S., Steinberger J. (2006). Cardiovascular risk reduction in high-risk pediatric patients: A scientific statement from the American Heart Association expert panel on population and prevention science; the councils on cardiovascular disease in the young, epidemiology and prevention. Circulation.

[118] Murphy E. (2011). Estrogen signaling and cardiovascular disease. Circ. Res..

[119] Ueda K., Karas R.H. (2013). Emerging evidence of the importance of rapid, non-nuclear estrogen receptor signaling in the cardiovascular system. Steroids.

[120] Pugach E.K., Blenck C.L., Dragavon J.M., Langer S.J., Leinwand L.A. (2016). Estrogen receptor profiling and activity in cardiac myocytes. Mol. Cell. Endocrinol..

[121] Ortona E., Gambardella L., Barbati C., Malorni W. (2014). Membrane-associated functional estrogen receptors alpha are upregulated in cardiomyocytes under oxidative imbalance. IJC Metab. Endocrinol..

[122] Matarrese P., Colasanti T., Ascione B., Margutti P., Franconi F., Alessandri C., Conti F., Riccieri V., Rosano G., Ortona E. (2011). Gender disparity in susceptibility to Oxidative stress and apoptosis induced by autoantibodies specific to RLIP76 in vascular cells. Antioxid. Redox Signal..

[123] Wang Y., Tuomilehto J., Jousilahti P., Antikainen R., Mähönen M., Katzmarzyk P.T., Hu G. (2011). Lifestyle factors in relation to heart failure among Finnish men and women. Circ. Heart Fail..

